# Collider and reporting biases involved in the analyses of cause of death associations in death certificates: an illustration with cancer and suicide

**DOI:** 10.1186/s12963-023-00320-y

**Published:** 2023-12-14

**Authors:** Moussa Laanani, Vivian Viallon, Joël Coste, Grégoire Rey

**Affiliations:** 1 French Centre for Epidemiology on Medical Causes of Death (CépiDc-Inserm), Le Kremlin-Bicêtre, France; 2 Biostatistics and Epidemiology Department, Cochin Hospital, Assistance Publique – Hôpitaux de Paris (AP-HP), Paris Cité University, Paris, France; 3 Strategy and Research Department, French National Health Insurance, Paris, France; 4 Nutritional Methodology and Biostatistics Group (NMB), International Agency for Research on Cancer (IARC) – World Health Organization, Lyon, France; 5 French National Public Health Agency, Saint-Maurice, France

**Keywords:** Causes of death, Causal inference, Collider bias, Reporting bias

## Abstract

**Background:**

Mortality data obtained from death certificates have been studied to explore causal associations between diseases. However, these analyses are subject to collider and reporting biases (selection and information biases, respectively). We aimed to assess to what extent associations of causes of death estimated from individual mortality data can be extrapolated as associations of disease states in the general population.

**Methods:**

We used a multistate model to generate populations of individuals and simulate their health states up to death from national health statistics and artificially replicate collider bias. Associations between health states can then be estimated from such simulated deaths by logistic regression and the magnitude of collider bias assessed. Reporting bias can be approximated by comparing the estimates obtained from the observed death certificates (subject to collider and reporting biases) with those obtained from the simulated deaths (subject to collider bias only). As an illustrative example, we estimated the association between cancer and suicide in French death certificates and found that cancer was negatively associated with suicide. Collider bias, due to conditioning inclusion in the study population on death, increasingly downwarded the associations with cancer site lethality. Reporting bias was much stronger than collider bias and depended on the cancer site, but not prognosis.

**Results:**

The magnitude of the biases ranged from 1.7 to 9.3 for collider bias, and from 4.7 to 64 for reporting bias.

**Conclusions:**

These results argue for an assessment of the magnitude of both collider and reporting biases before performing analyses of cause of death associations exclusively from mortality data. If these biases cannot be corrected, results from these analyses should not be extrapolated to the general population.

**Supplementary Information:**

The online version contains supplementary material available at 10.1186/s12963-023-00320-y.

## Background

National cause of death data are widely used to describe the health of populations [1]. These data are exhaustive and collected in a standardised fashion, allowing international comparisons [2]. They are extracted from medical death certificates where certifiers (physicians or coroners) are asked to describe the causal sequence leading to death. These data have been studied to assess associations between diseases in the general population [3–9], although the difficulties of such study design have long been emphasised [10–12]. For example, the risk of suicide in patients with Parkinson’s disease was estimated in an often-cited study based on death certificate data [5]. The authors found a tenfold lower risk of suicide in people with Parkinson’s disease than for other individuals who died. However, instead of a decreased risk, prospective studies highlighted a two- to fivefold higher suicide risk in these patients [13, 14]. Indeed, the design used in the first study (estimating associations between health states in the general population from mortality data) is subject to two main types of bias, which could explain misleading findings. Another interesting example is that of a study conducted by an Australian team on multiple causes of death data, in which the authors assessed the prevalence of mental and physical diseases in suicide decedents as compared with the general population [15]. Considering that the whole population of non-suicide decedents was not representative of the whole living population, they compared suicide decedents with accident decedents. They found an increased risk of suicide associated with cancer, but a strongly decreased risk of suicide with other somatic diseases. This study was reproduced shortly afterwards by an American team, with consistent results [6]. However, more recent studies, based on data on the whole living population, did not confirm the strongly reduced risk of suicide associated with non-cancer physical diseases [16–19], suggesting that some amount of bias remains when assessing associations of disease states in the general population comparing restricted groups of causes of death in multiple causes of death studies.

### Collider bias

**Fig. 1:**
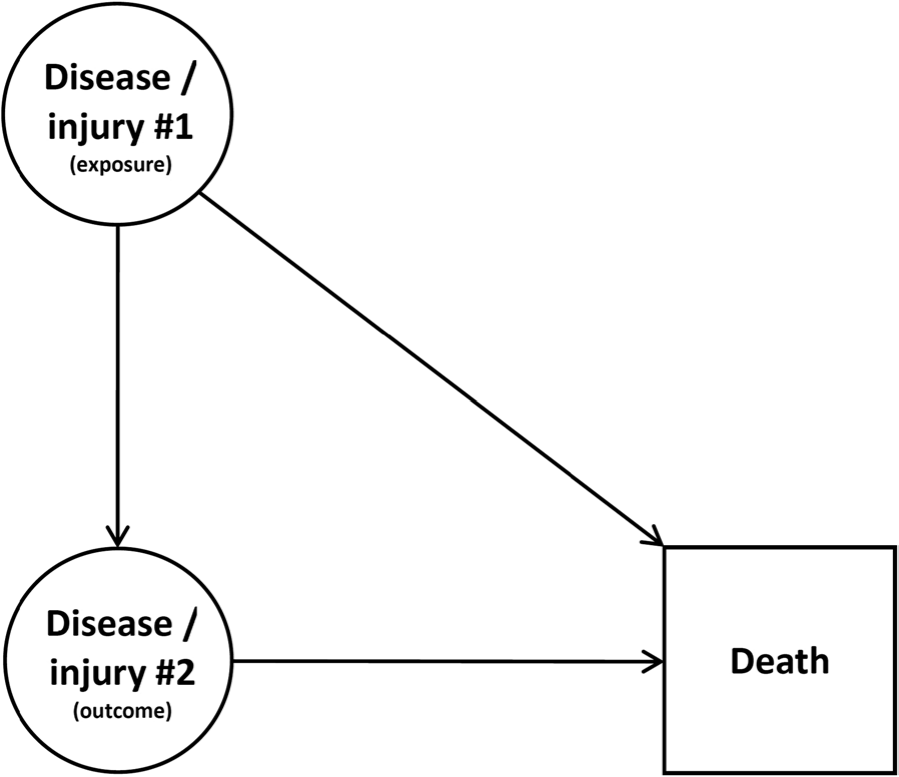
Directed acyclic graph representing the causal process underlying studies based on causes of death data. Collider bias emerges from conditioning inclusion in the study population on death, which is a consequence (descendant) of both exposure and outcome, which are the two factors defined among the diseases and injuries coded as causes of death for which the association is assessed. In our illustrative example, cancer is “disease #1” (the exposure), and suicide is “injury #2” (the outcome). Death is the collider on which inclusion in the study population is conditioned

Studies based on death certificate data are conducted on non-representative samples of the general population. Indeed, even if all deaths are reported, no information is available on living individuals. This leads to a selection bias, as inclusion in the study population is conditioned on death, which is a common effect of the diseases under study (defined among causes of death), called a collider (Fig. 1). This selection bias is called “collider bias”, or “bias due to conditioning on a collider” and can strengthen or reverse associations between variables of interest [20, 21].

### Reporting bias

Studies on death certificate data are also subject to measurement error or information bias [10], which we hereafter refer to as “reporting bias”. This bias, which can be differential (depending on the value of other variables under study) or non-differential, may result from (1) the requested task assigned to the certifier, who has to report diseases and events that effectively contributed to death only, rather than all diseases present prior to death that contribute to the poor health state, and (2) possible incompleteness in the filling out of the death certificates (which depends, among other things, on the certifier’s level of knowledge of the deceased patient and his/her medical history) [22].

### Aim and organisation of the paper

Seminal literature that warned on the risks of using comprehensive mortality data to assess associations between diseases only provided leads to reduce these risks, without giving a deep insight into the mechanisms of the biases involved [10]. The general purpose of this paper was to assess to what extent associations of causes of death estimated from individual mortality data can be extrapolated as associations of disease states in the general population, given collider and reporting biases. As an illustrative example, we estimated the association between cancer and suicide in death certificates depending on the cancer site and assessed the order of magnitude of the collider and reporting biases. In the first section of the paper, we describe how multiple causes of death data are constructed, from medical certification to medical coding of causes of death (including the international rules for the selection of the underlying cause of death). We also describe the framework for the assessment of associations between causes of death and the biases involved in such studies. In the second section, we present the methods and results of our illustrative example on cancer and suicide. Finally, we conclude by addressing recommendations for future studies and discussing how to improve the use of multiple causes of death data.

## Analyses of cause of death associations in death certificates

### Mortality data obtained from death certificates

**Fig. 2:**
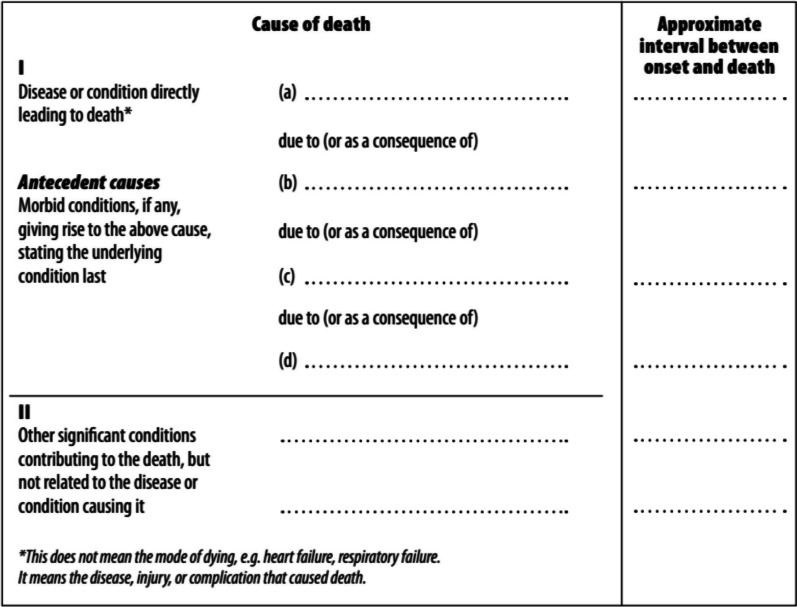
International form of medical certificate of cause of death (WHO, ICD-10, 1993)

Medical certification of death is mandatory in most industrialized countries and must be performed by a physician or a coroner. The World Health Organization (WHO) has designed the structure of the international medical death certificate with two parts: Part I is dedicated to the description of the causal sequence of events that directly led to death and Part II the reporting of significant morbid conditions that may have contributed to death but are not involved in the sequence of events that directly led to death (Fig. 2).

The WHO defines the *underlying cause of death* as “the disease or injury which initiated the train of morbid events leading directly to death or the circumstances of the accident or violence which produced the fatal injury” [23]. Selection of the underlying cause of death is performed automatically by software (e.g. Iris) [24] or based on the expertise of a mortality medical coder (or “nosologist”) for the most complex cases. This selection is governed by several rules prescribed by the WHO in the tenth revision of the International Statistical Classification of Diseases and Related Health Problems [ICD-10] [23]. The main rule, called the “General Principle”, states that “when more than one condition is entered on the certificate, […] the condition entered alone on the lowest used line of Part I” (i.e. the first condition mentioned in the train of morbid events leading to death) must be selected as the underlying cause of death, “only if it could have given rise to all the conditions entered above it” (i.e. to the subsequent conditions of the train of morbid events leading to death) [23]. If the General Principle does not apply, Rules 1 and 2 state that the originating cause of the immediate (or final) cause of death, mentioned first in the train of morbid events leading to death, has to be selected as the underlying cause of death. Finally, Rule 3 states that “if the condition selected by the [previous rules] was obviously a direct consequence of another reported condition, whether in Part I or Part II”, this condition has to be selected as the underlying cause of death [23]. For instance, HIV disease and external causes of death can meet Rule 3.

### Framework for the assessment of associations between causes of death and the biases involved

Mortality data can be used to assess associations between health states (diseases and/or injuries) mentioned as causes of death. Standardised mortality ratios are a tool to assess such associations [10, 25]. Multivariable logistic regression models can also be used, allowing adjustment for potential confounders. Odds ratios [OR], resulting from these models, convey information concerning both the direction of the association (the risk is higher if OR > 1 or lower if OR < 1) and its magnitude. When the prevalence of the assessed outcome is low, the OR is a good approximation of the relative risk and can be interpreted accordingly [10].

#### Assessment of collider bias

Collider bias is due to conditioning the study sample on death. A multistate model can be used to generate populations of individuals and simulate their health states up to their deaths from national health statistics. Associations between health states can then be estimated from such simulated deaths (with logistic regression models, in the same way as with observed deaths) and the collider bias assessed, as these simulated deaths artificially replicate this bias. Collider bias can then be estimated from the following ratio:

$${\text{Collider}}\;{\text{bias}} = \frac{{{\text{Real}}\;\left( {{\text{unbiased}}} \right)\;{\text{association}}\;{\text{measure}}}}{{{\text{Association}}\;{\text{measure}}\;{\text{estimated}}\;{\text{on}}\;{\text{the}}\;{\text{simulated}}\;{\text{deaths}}}}.$$

Multiplicative measures of bias are better suited in this context, in which associations are expressed in the multiplicative scale (ORs).

#### Assessment of reporting bias

The magnitude of reporting bias can be approximated by the difference between the estimates obtained from observed death certificates (which are subject to both collider and reporting biases) and those obtained from simulated deaths (which are subject to collider bias only). Reporting bias can then be approximated from the following ratio:

$${\text{Reporting}}\;{\text{bias}} = \frac{{{\text{Association}}\;{\text{measure}}\;{\text{estimated}}\;{\text{on}}\;{\text{the}}\;{\text{simulated}}\;{\text{deaths}}}}{{{\text{Association}}\;{\text{measure}}\;{\text{estimated}}\;{\text{on}}\;{\text{the}}\;{\text{observed}}\;{\text{deaths}}}}.$$

However, the two sources of reporting bias ((1) the difference of the definition between measuring associations of diseases and measuring associations of causes of death and (2) the incomplete filling out of death certificates by certifiers) cannot be distinguished from one another.

## Illustrative example: association between cancer and suicide in death certificates in France

Suicide is a major public health issue, accounting for 1.4% of all deaths worldwide [26]. The impact of psychiatric diseases (notably, depression, anxiety, and psychotic disorders) [27] is well known, but somatic disorders may also play a role in the occurrence of suicide deaths. Cancer, due to its impact on health, the adverse events of treatments, and stigma, can substantially reduce the quality of life and promote the onset of suicidal ideation and suicide deaths. This phenomenon can vary depending on the cancer site prognosis, notably after receiving the diagnosis [28].

Our illustrative example is based on French multiple causes of death data. Mortality data are commonly used to study suicide mortality and its determinants, with various study designs: ecological studies [29], studies based on disease registries [30], analyses of cause of death associations [5, 6]. Inclusion in our study population was structurally conditioned on death, a common effect of cancer (the exposure) and suicide (the outcome), i.e. a collider (Fig. 1). We first measured the cancer/suicide association in death certificates, according to cancer site, and then assessed the magnitude of the collider and reporting biases, using simulations.

### Methods

#### French mortality data

All deaths of people aged 15 years or older occurring in mainland France between 2000 and 2013 were included in the study, provided that at least one cause was mentioned. Causes of death were coded (throughout the study period) according to the ICD-10 [23]. Suicide (ICD-10 codes: X60 to X84 and Y87.0) was defined from the underlying causes of death, as suicide meets Rule 3 criteria: wherever "suicide" is mentioned on the death certificate, it is almost always selected as the underlying cause of death, even if the certifier indicated that suicide was secondary to depressive disorders or cancer. Cancer (ICD-10 codes: C, see the list of cancer sites in Additional file 1: Table S1) was defined from both the underlying cause of death and Part II diagnoses; if cancer was not the first cause in the train of morbid events leading to death declared by the certifier in Part I of the death certificate, it was sought among all other diagnoses, except those mentioned between the immediate cause of death and the underlying cause of death selected by following WHO rules, considered to be consequences of the underlying cause of death. This type of situation is relatively uncommon and concerns exclusively cancer associated with HIV/AIDS [23]. Such a focus on the first cancer site mentioned in the train of morbid events leading to death prevents consideration of secondary cancer sites (including metastases).

#### Simulation scheme

We performed a simulation study to assess the direction and magnitude of the collider bias involved in this illustrative example. A population of 5 million women and 5 million men was generated using national statistics of mortality and cancer incidence to simulate the occurrence of cancer as well as death from cancer, suicide, and other causes. Focusing on deaths occurring between 15 and 110 years old, we studied the association between cancer and suicide in the corresponding death certificates to ascertain the presence and magnitude of collider bias. A first simulation study was conducted under the null hypothesis of no cancer/suicide association (i.e. in which the transition probability from a *K*th cancer state to the suicide death state equals that from the healthy state to the suicide death state) to assess whether collider bias alone could induce high amplitude false associations and determine the direction of such bias. A second simulation study was conducted to approximate the magnitude of the collider bias, using approximations of the real cancer/suicide associations in the French population. To do so, we used relative risks of suicide death for several cancer sites estimated in a recent large cohort study conducted from national Swedish registers (Fang et al.’s study) [28].

**Fig. 3:**
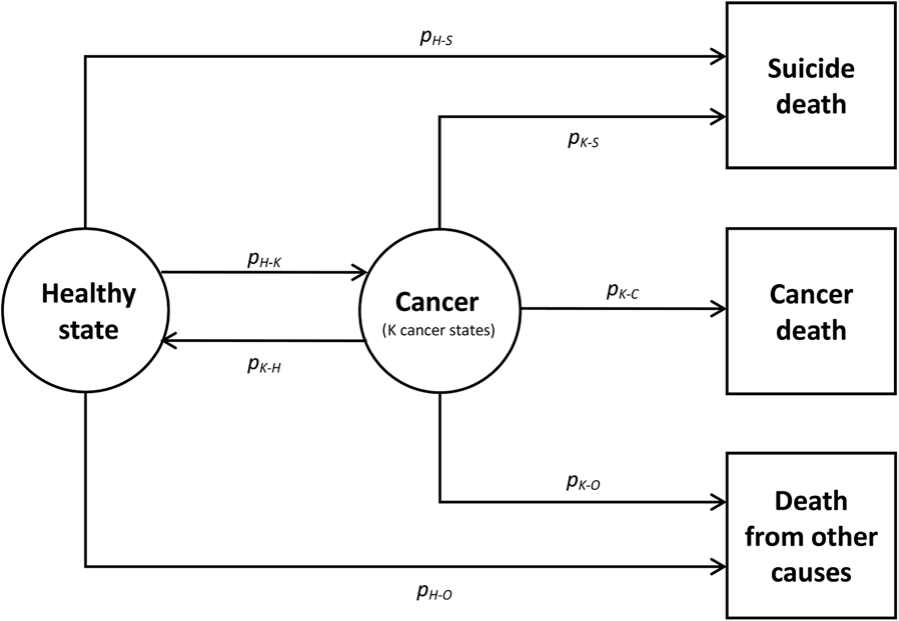
Multistate model used for the simulation of death data in people aged 15 years or older. Transition probabilities were obtained from national cancer incidence and survival and suicide mortality statistics: *p*_H–S_ = transition probability from the initial healthy state to the absorbing suicide death state, *p*_H–K_ = transition probabilities from the initial healthy state to the *K*th cancer state, *p*_H–O_ = transition probability from the initial healthy state to the absorbing other causes of death state, *p*_K–H_ = transition probabilities from the *K*th cancer state to the initial healthy state, *p*_K–S_ = transition probabilities from the *K*th cancer state to the absorbing suicide death state, *p*_K–C_ = transition probabilities from the *K*th cancer state to the absorbing *K*th cancer death state, *p*_K–O_ = transition probabilities from the *K*th cancer state to the absorbing other causes of death state. The probability of suicide death for individuals in a *K*th cancer state *p*_K–S_ was obtained by multiplying the relative risk of suicide corresponding to the *K*th cancer site by the national suicide mortality rate. In the first simulation study, the relative risks of suicide used were equal to one for every cancer site (to mimic the null hypothesis of no cancer/suicide association); in the second simulation study, those published in the study of Fang et al. were applied [28]

Deaths from suicide, cancer, and other causes for people aged 15 years or older were simulated using a multistate model, with deaths as absorbing states (Fig. 3). Transition probabilities to move from one state to another within a year were functions of age and gender. Simulations were performed separately for each gender. Individuals entered the model at age 15 years in the initial healthy state. Individuals could then transit to one of the *K* cancer states (for *K* cancer sites listed in Additional file 1: Table S1) or die from suicide or other causes. Once in one of the *K* cancer states, individuals could die from the *K*th cancer, suicide, or other causes, or go back to the healthy state if they did not die within five years. Transition probabilities were derived from national suicide mortality [31] and cancer incidence [32] and survival [33] statistics. Considering individuals in a *K*th cancer state, net survival was used as the probability of death from the *K*th cancer and the difference between net and crude survival as the probability of death from other causes [33]. The probability of suicide death for individuals in a *K*th cancer state was obtained by multiplying the relative risk of suicide corresponding to the *K*th cancer site by the national suicide mortality rate. In the first simulation study, the relative risks of suicide used were equal to one for every cancer site (to mimic the null hypothesis of no cancer/suicide association); in the second simulation study, those published in the study of Fang et al. were applied [28]. For cancer sites not assessed in their study, the mean relative risk of suicide for other cancer sites was used. The simulations were performed using R (V3.4.0) [34].

#### Statistical analyses

Associations between cancer sites and suicide were estimated for both observed and simulated deaths, with logistic regression models adjusted for age (B-spline with 3 degrees of freedom), gender, and, for observed data, region of death. Analyses were conducted for both genders together for the cancer sites studied by Fang et al., and, in complementary analyses, for men and women separately for the cancer sites listed in Additional file 1: Table S1, as both cancer epidemiology and suicide epidemiology differ according to gender [35].

The direction of collider bias was determined using the ORs obtained from the first simulation study (under the null hypothesis). If the OR obtained in the first simulation was lower than 1, the direction of collider bias was considered to be negative, whereas it was considered to be positive if it was higher than 1. If the OR obtained equalled 1, then it was considered that there was no collider bias.

The magnitude of the collider bias was assessed using the second simulation. In the absence of collision, ORs obtained in the second simulation study should be similar to those reported by Fang et al. Indeed, if no collider bias was involved in this simulation study, the input used to determine the transition probabilities from a cancer state to the suicide death state (i.e. the relative risk of suicide from the study of Fang et al.) should have been found. The magnitude of collider bias was then evaluated by computing the ratio between the relative risk of suicide from the study of Fang et al. and the OR estimated from the second simulation. As suicide deaths occur rarely in the population, OR and relative risk values can be considered to be relatively similar (approximately 1 death out of 60 is suicide in the French population).

The magnitude of the reporting bias was evaluated by comparing the OR estimated from the second simulation and that estimated from observed death certificates. Under the assumptions that our simulations correctly reproduced the French mortality data, that the cancer/suicide associations found by Fang et al. are close to those existing in the French population, and that there are no remaining confounders, differences between the results obtained using the data from the second simulation study and the observed deaths are likely to be largely attributable to reporting bias.

Statistical analyses were performed using SAS version 9.4 (SAS Institute, Cary, North Carolina) [36].

### Results

#### French mortality data

Overall, 7.2 million deaths between 2000 and 2013 were considered (3,685,024 of men, of which 107,241 were suicides (3%), and 3,553,707 of women, of which 38,297 were suicides (1%)). The number of deaths (suicide or other causes) according to the presence or not of a cancer diagnosis among causes of death are detailed in Table 1. The analyses performed on mortality data showed a highly negative association between suicide and each cancer site (OR adjusted for age, gender, and region of death ranged from 0.01 for central nervous system cancer and cutaneous melanoma, 95% confidence intervals (95% CI) [0.01–0.01] and [0.01–0.02], respectively, to 0.24 for prostate cancer, 95% CI = [0.22–0.26]; see Table 2). The study of Fang et al. found a positive association between suicide and each cancer site (with adjusted relative risk from 1.4 for cutaneous melanoma to 4.5 for oesophageal, liver, and pancreatic cancer) (Table 2). Our results were thus inconsistent with theirs.

**Table 1:** Characteristics of the study population (mortality data observed from death certificates, France, 2000–2013)

Gender	Cause of death		Number (%)	Age at death, median [IQR]
Men	Overall		3,685,024 (100)	76 [64–84]
	Suicide	Bladder cancer	150 (0)	78 [69–83]
		CNS cancer	20 (0)	56 [45–64]
		Colorectal cancer	331 (0)	75 [68–81]
		Cutaneous melanoma	13 (0)	76 [58–80]
		Head and neck cancer	157 (0)	65 [55–75]
		Kidney cancer	77 (0)	71 [65–77]
		Larynx cancer	103 (0)	67 [57–75]
		Liver cancer	82 (0)	72 [63–76]
		Lung cancer	448 (0)	70 [62–77]
		Oesophageal cancer	104 (0)	71 [61–79]
		Pancreatic cancer	110 (0)	73 [65–80]
		Prostate cancer	643 (0)	79 [72–84]
		Stomach cancer	100 (0)	75 [65–81]
		Testis cancer	9 (0)	52 [44–55]
		Thyroid gland cancer	6 (0)	76 [47–78]
		Other or no cancer^a^	104,888 (2.9)	50 [39–66]
	Other cause	Bladder cancer	53,867 (1.5)	77 [69–84]
		CNS cancer	24,204 (0.7)	64 [53–73]
		Colorectal cancer	130,632 (3.5)	76 [67–82]
		Cutaneous melanoma	12,298 (0.3)	68 [56–78]
		Head and neck cancer	46,689 (1.3)	62 [54–71]
		Kidney cancer	30,515 (0.8)	73 [63–81]
		Larynx cancer	19,431 (0.5)	66 [57–76]
		Liver cancer	80,578 (2.2)	71 [63–78]
		Lung cancer	309,349 (8.4)	68 [59–77]
		Oesophageal cancer	45,215 (1.2)	68 [58–77]
		Pancreatic cancer	61,217 (1.7)	71 [62–79]
		Prostate cancer	154,378 (4.2)	82 [75–87]
		Stomach cancer	43,501 (1.2)	74 [64–81]
		Testis cancer	1503 (0)	45 [33–63]
		Thyroid gland cancer	2227 (0.1)	72 [62–80]
		Other or no cancer^a^	2,562,179 (70)	78 [67–86]
Women	Overall		3,553,707 (100)	84 [76–90]
	Suicide	Bladder cancer	10 (0)	76 [57–87]
		Breast cancer	264 (0)	63 [52–74]
		CNS cancer	4 (0)	62 [50–77]
		Colorectal cancer	53 (0)	73 [63–83]
		Corpus uteri cancer	7 (0)	70 [62–77]
		Cutaneous melanoma	6 (0)	77 [74–79]
		Head and neck cancer	17 (0)	61 [57–76]
		Kidney cancer	11 (0)	73 [62–81]
		Larynx cancer	4 (0)	54 [50–59]
		Liver cancer	4 (0)	68 [63–72]
		Lung cancer	37 (0)	67 [56–78]
		Oesophageal cancer	10 (0)	73 [51–80]
		Ovary cancer	8 (0)	66 [58–70]
		Pancreatic cancer	19 (0)	71 [68–81]
		Stomach cancer	9 (0)	64 [53–70]
		Thyroid gland cancer	5 (0)	79 [67–81]
		Other or no cancer^a^	37,829 (1.1)	53 [42–68]
	Other cause	Bladder cancer	16,694 (0.5)	82 [75–88]
		Breast cancer	180,821 (5.1)	74 [60–84]
		CNS cancer	18,831 (0.5)	68 [57–77]
		Colorectal cancer	116,793 (3.3)	80 [71–87]
		Corpus uteri cancer	10,105 (0.3)	75 [67–82]
		Cutaneous melanoma	10,472 (0.3)	73 [57–83]
		Head and neck cancer	9,737 (0.3)	67 [56–80]
		Kidney cancer	17,161 (0.5)	78 [69–85]
		Larynx cancer	2,125 (0.1)	67 [57–79]
		Liver cancer	28,184 (0.8)	77 [69–84]
		Lung cancer	89,479 (2.5)	69 [57–79]
		Oesophageal cancer	10,597 (0.3)	75 [63–83]
		Ovary cancer	47,964 (1.4)	73 [63–81]
		Pancreatic cancer	57,766 (1.6)	78 [69–84]
		Stomach cancer	25,717 (0.7)	80 [70–87]
		Thyroid gland cancer	3,896 (0.1)	79 [71–86]
		Other or no cancer^a^	2,869,068 (81)	86 [79–91]

*CNS* Central nervous system, *IQR* Interquartile range

^a^Includes other cancer sites, multiple cancers, and haematological malignancies

**Table 2:** Suicide ORs by cancer site in observed and simulated mortality data, and estimated bias magnitudes

Cancer site	French mortality data		Simulation #1: independence		Simulation #2: RR from Fang et al. [4]		Fang et al. study[4]	Collider bias^b^	Reporting bias^c^
	OR	[95% CI]	OR	[95% CI]	OR	[95% CI]	RR		
No cancer^a^	1.00		1.00		1.00		1.0	1.0	1.0
Prostate	0.24	[0.22;0.26]	0.71	[0.68;0.75]	1.14	[1.10;1.18]	1.9	1.7	4.7
Cutaneous melanoma	0.01	[0.01;0.02]	0.61	[0.53;0.70]	0.77	[0.68;0.86]	1.4	1.8	64
Breast	0.04	[0.03;0.04]	0.58	[0.52;0.64]	0.85	[0.78;0.92]	1.6	1.9	24
Colorectal	0.05	[0.05;0.06]	0.35	[0.33;0.37]	0.50	[0.48;0.52]	1.6	3.2	9.4
Oesophageal, liver, pancreatic	0.03	[0.03;0.03]	0.17	[0.16;0.19]	0.55	[0.53;0.58]	4.5	8.1	20
Lung	0.02	[0.02;0.02]	0.14	[0.13;0.15]	0.36	[0.35;0.38]	3.3	9.1	17
Central nervous system	0.01	[0.01;0.01]	0.11	[0.09;0.14]	0.25	[0.22;0.28]	2.3	9.3	35

Logistic regression models adjusted for age (B-spline of degree 3), gender, and region of death; mainland France, 2000–2010

*OR* Odds ratio, *RR* Relative risk, *95% CI* 95% Confidence interval

^a^Excluding other cancer sites, multiple cancers, and haematological malignancies. The magnitude of the biases was estimated from the following ratios

^b^Collider bias = RR from Fang et al./OR estimated from the data of simulation #2

^c^Reporting bias = OR estimated from the data of simulation #2/OR estimated from observed deaths

#### Estimation of the magnitude of the biases

Each simulation generated 4.7 million deaths for men, of which 2% were suicides, and 4.6 million for women, of which 1% were suicides. The proportion of deaths due to each cause and age distributions at death were similar between the simulated data and that from mortality data (Additional file 1: Table S2). The first simulation study, conducted under the null hypothesis of no cancer/suicide association, found a negative association for each cancer site, with OR ranging from 0.11 (95% CI = [0.09–0.14]) for central nervous system cancer to 0.71 (95% CI = [0.68–0.75]) for prostate cancer. In the absence of collision, these ORs were expected to be 1 for all cancer sites. The results were thus biased downward by collision.

The second simulation (conducted using the relative risks of suicide published by Fang et al.) [28] found a negative cancer/suicide association for all cancer sites (OR from 0.25, 95% CI = [0.22–0.28], for central nervous system cancer to 0.85, 95% CI = [0.78–0.92], for breast cancer), except for prostate cancer (OR = 1.14, 95% CI = [1.10–1.18]), although it was lower than the relative risk reported by Fang et al. (1.9). In the absence of collider bias, these ORs were expected to be similar to those published by Fang et al.

**Fig. 4:**
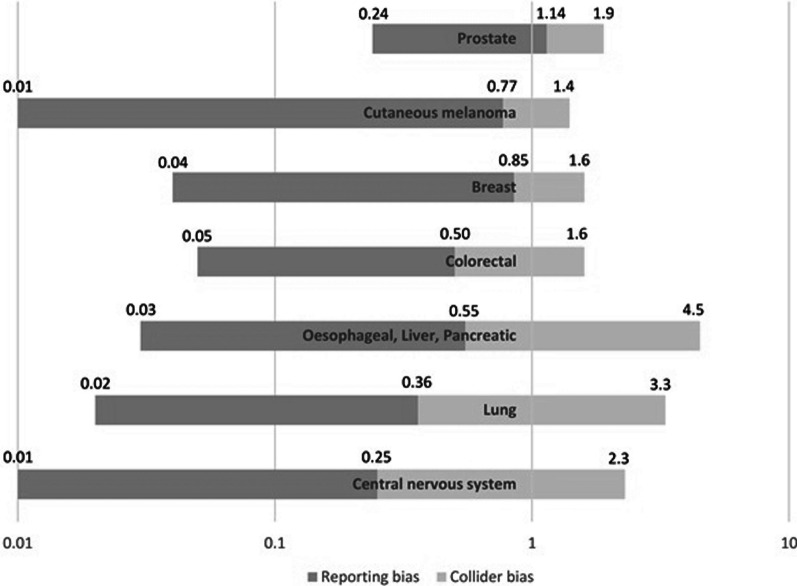
Magnitude of collider and reporting biases, according to cancer site. The figure is interpreted as follows: The unbiased relative risk (approximated from that of the study of Fang et al.) is at the right end of the bar. The light grey part of the bar represents the magnitude of the collider bias. The odds ratio from simulation #2 is at the junction between the light and the dark grey parts of the bar. The dark grey part of the bar represents the magnitude of the reporting bias. The observed odds ratio (obtained from French mortality data) is at the left end of the bar. For example, for breast cancer, the unbiased relative risk of suicide is 1.6. The collider bias divides this relative risk by 1.9. The odds ratio from simulation #2 is 0.85. The reporting bias divides this odds ratio by 24. The odds ratio observed from French mortality data are 0.04. The scale of the *x*-axis is logarithmic

Collider bias was estimated to divide the relative risk of suicide reported by Fang et al. by at least 1.7 (for prostate cancer) and up to 9.3 (for central nervous system cancer). The magnitude of collider bias thus varied according to cancer site and appeared to increase with cancer site lethality, as expected. Estimating collider bias from simulation #1 (with the inverse of the obtained OR) produced consistent results. Reporting bias was found to divide the OR of suicide from the second simulation (i.e. the relative risk of Fang et al. biased by collision) by at least 4.7 (for prostate cancer) and up to 64 (for cutaneous melanoma). Using our approximation, the magnitude of reporting bias was thus much higher than that of collider bias. The magnitude also varied depending on the cancer site, but did not appear to be associated with cancer site lethality. The magnitudes of the collider and reporting biases are presented in Fig. 4.

Complementary analyses performed for each gender separately gave similar results for men (Additional file 1: Table S3). The results were slightly different for women, with a higher overall magnitude of bias. We found the lowest magnitude for collider bias for cutaneous melanoma and the highest for lung cancer, and the lowest magnitude for reporting bias for oesophageal cancer and the highest for liver cancer (Additional file 1: Table S4).

## Discussion

Here, we demonstrated that estimating associations between diseases from mortality data (i.e. from death certificate data) is exposed to biases and used an illustrative example to assess their direction and magnitude. The cancer/suicide association was inverse when assessed based on mortality data (OR ranging from 0.24 for prostate cancer to 0.01 for central nervous system cancer and cutaneous melanoma). However, previous longitudinal studies found positive associations, as notably reported by Fang et al., who found a relative risk of suicide that ranged from 1.4 to 4.5, depending on the cancer site [28]. Part of this discrepancy is attributable to collider bias, which naturally arises when cancer/suicide associations are assessed from mortality data [20, 21]. We performed simulations to artificially reproduce collider bias by generating deaths from national statistics of suicide and cancer incidence and mortality. Analyses performed on such simulated deaths showed that conditioning inclusion in the study population on death biased the results towards negative associations, the bias increasing with cancer site lethality. However, such collider bias was not sufficient to fully explain the discrepancies between the results based on death certificates and those reported by Fang et al. Although there are other potential explanations (the two source populations differed, as the study of Fang et al. was performed in Sweden), we believe that the remaining bias can be largely attributed to reporting bias [22, 37]. Our approximation of reporting bias was much stronger than collider bias and depended on the cancer site, but not the prognosis, as the magnitude of the reporting bias varied between cancer sites, but not according to cancer lethality.

### Biases involved in the analyses of cause of death associations in death certificates

Collider bias was first described recently [38] and is of increasing concern among epidemiologists. This type of selection bias has been the source of much scientific debate, such as for the so-called “birth weight paradox”. Let us consider, for example, the risk of neonatal death associated with maternal smoking, which is known to increase the risks of both low birth weight and neonatal mortality. Comparing mortality rates between low birth weight infants born to smokers and those born to non-smokers paradoxically lead to finding lower mortality rates in infants of smokers [39]. Such results “raised doubts” about the pejorative impact of maternal smoking [40]. However, this paradox may be explained by collider bias, as demonstrated by Hernández-Díaz et al. [41]. Indeed, low birth weight is a collider on which selection in the study sample is conditioned, as it is a common effect of maternal smoking and other unmeasured causes (such as birth defects or malnutrition). The “obesity paradox” is another example of a scientific controversy that may be explained by collider bias. This paradox refers to the lower mortality observed for obese patients, found, for example, among patients with diabetes [42–45]. Collider bias should be considered in all studies conducted with a case-only design [21], notably those analysing associations of causes of death from mortality data. To our knowledge, our study is the first to consider collider bias in this specific type of studies.

Interpreting reporting bias is challenging and requires consideration of its two sources. This type of information bias is due (1) to the difference between what is asked of the certifier (i.e. reporting a causal sequence of injuries and diseases leading to death) and the information that would be expected for epidemiology (i.e. diseases reported regardless of their potential causal link with death) [23]. This specificity gives multiple causes of death databases their particular interest as they thus provide the opportunity to assess causal relations between diseases or morbid conditions. In return, the information available in causes of death data is very conservative. Reporting bias is also due (2) to the incompleteness of certificate filling by certifiers. This depends on the certifier’s knowledge of the deceased patient’s medical history and knowledge (or intuition) of the possibility of a causal association between the underlying cause of death and its comorbidities [46, 47]. In our application, without knowledge/intuition of the plausible link between one’s cancer and suicide, the certifier might not mention cancer on the certificate.

Unmeasured confounding is a source of bias we did not address in this paper [48]. We rather focussed on collider and reporting bias for pedagogical reasons to correctly identify them. Unmeasured confounding is often involved when one wants to estimate causal effects. We aimed in our illustrative example to compare our associational ORs with the associational risk ratios of Fang et al. In this situation, confounding may be considered to be negligible and is essentially amongst the supplementary factors for which Fang et al. adjusted their models [28]. Both our study and that of Fang et al. adjusted for age and gender, which are major confounders in the cancer/suicide association. Fang et al. also adjusted their models for cohabitation status, socioeconomic status, and educational level, but did not adjust for other major confounders in the cancer/suicide association, such as alcohol consumption [27, 49].

## Conclusions

While risks of using comprehensive mortality data to assess associations between diseases have long been highlighted [10–12], our work aimed to explain the mechanisms of the biases involved in such studies. We used a conceptual framework to demonstrate the impossibility of measuring causal associations from multiple causes of death data. We used a simulation study to assess the magnitude of the involved biases, accounting for the specificities of death certificates. Even if we could have tried to correct for collider bias in our illustrative example (by an indicator of cancer site prognosis, such as survival rate), our results show that reporting bias was of much higher magnitude and heterogeneous across cancer sites. Reporting bias cannot be corrected, as the reason for such heterogeneity could not be clearly linked with the cancer site characteristics. In analyses of cause of death associations exclusively from mortality data (i.e. from death certificates), if the reporting bias is too strong, there is little use in correcting for collider bias and results from these analyses should not be extrapolated to the general population. Multiple causes of death data are still a remarkably rich source because of their standardised construction and international comparability and because they contain directed causal information, integrating the expert knowledge of the physician or coroner certifying death. Given the impact of collider and reporting biases, the analyses of these data should not be considered valid when conducted as in this paper. They should be performed after full linkage to comprehensive databases, such as registers or medical administrative databases, to take full advantage of these qualities and avoid drawing conclusions based on spurious associations [16, 50, 51]. The issue raised here regarding collider bias can be extended to other case-only designs [21], including studies on pharmacovigilance databases or disease registries; reporting bias issues are specific to each data type.

### Supplementary Information

**Additional file 1: Table S1.** ICD-10 codes used to define cancer. **Table S2.** Characteristics of the simulated populations. **Table S3.** Suicide ORs by cancer site in men in observed and simulated mortality data and estimated bias magnitudes. **Table S4.** Suicide ORs by cancer site in women in observed and simulated mortality data and estimated bias magnitudes.

## Data Availability

Anonymized individual data from death certificates used in this work can be shared by the authors under strict security conditions. Applications to access these French mortality data must be submitted to the French Health Data Hub (https://www.health-data-hub.fr/). Aggregated data used for the simulation study are publicly available: National suicide mortality rates are available in the CépiDc (French Centre for Epidemiology on Medical Causes of Death) repository (http://www.cepidc.inserm.fr/inserm/html/index2.htm [31]), Cancer incidence and survival rates are available in the SPF (Public Health France) repository (http://invs.santepubliquefrance.fr/Dossiers-thematiques/Maladies-chroniques-et-traumatismes/Cancers/Surveillance-epidemiologique-des-cancers/Estimations-de-l-incidence-de-la-mortalite-et-de-la-survie-stade-au-diagnostic [32, 33]), or on the INCa (French National Cancer Institute) repository (https://lesdonnees.e-cancer.fr/ [55]).

## Abbreviations

95% CI: 95% Confidence interval
CNS: Central nervous system
HIV/AIDS: Human immunodeficiency virus / acquired immune deficiency syndrome
ICD-10: Tenth revision of the International Statistical Classification of Diseases and Related Health Problems
IQR: Interquartile range
OR: Odds ratio
WHO: World Health Organization
