# Supplementary material for: Collider and reporting biases involved in the analyses of cause of death associations in death certificates: an illustration with cancer and suicide
Source: Popul Health Metr. 2023 Dec 14;21:21. doi: 10.1186/s12963-023-00320-y (PMC10722743; doi:10.1186/s12963-023-00320-y)
Supplement: Supplementary file 1 — Additional file 1: Table S1. ICD-10 codes used to define cancer. Table S2. Characteristics of the simulated populations. Table S3. Suicide ORs by cancer site in men in observed and simulated mortality data and estimated bias magnitudes. Table S4. Suicide ORs by cancer site in women in observed and simulated mortality data and estimated bias magnitudes. [file 12963_2023_320_MOESM1_ESM.docx]

**Collider and Reporting Biases Involved in the Analyses of Cause of Death Associations in Death Certificates: an Illustration with Cancer and Suicide**

# Additional material

**Additional table 1.** ICD-10 codes used to define cancer

**Additional table 2.** Characteristics of the simulated populations

**Additional table 3.** Suicide ORs by cancer site in men in observed and simulated mortality data, and estimated bias magnitudes

**Additional table 4.** Suicide ORs by cancer site in women in observed and simulated mortality data, and estimated bias magnitudes

**Additional table 1. ICD-10 codes used to define cancer**

| **Cancer site** | **ICD-10 codes** |
| --- | --- |
| **Bladder** | C67 |
| **Breast (female)** | C50 |
| **Central nervous system** | C70-C72 |
| **Colorectal** | C18-C21 |
| **Corpus uteri** | C54 |
| **Cutaneous melanoma^a^** | C43 |
| **Head and neck** | C01-C06, C09-C14 |
| **Kidney** | C64 |
| **Larynx** | C32 |
| **Liver^b^** | C22 |
| **Lung** | C33-C34 |
| **Oesophagus^b^** | C15 |
| **Ovary** | C56, C57.0-C57.4 |
| **Pancreas^b^** | C25 |
| **Prostate** | C61 |
| **Stomach** | C16 |
| **Testis** | C62 |
| **Thyroid gland** | C73 |

^a^ In Fang *et al*. study, this site was broadened to “skin cancer”.

^b^ Liver, oesophageal, and pancreatic cancers were pooled together as a group, as in the study of Fang *et al*.

**Additional table 2. Characteristics of the simulated populations**

|  |  |  | **Data from simulation #1** | | **Data from simulation #2** | |
| --- | --- | --- | --- | --- | --- | --- |
| **Gender** | **Cause of death** | | **Number (%)** | **Age at death, median [IQR]** | **Number (%)** | **Age at death, median [IQR]** |
| **Men** | Overall |  | 4,796,597 (100%) | 75 [58-84] | 4,760,806 (100%) | 75 [61-85] |
|  | Suicide | Bladder cancer | 215 (0%) | 68 [57-81] | 596 (0%) | 70 [59-83] |
|  |  | CNS cancer | 66 (0%) | 70 [56-87] | 164 (0%) | 70 [54-81] |
|  |  | Colorectal cancer | 887 (0%) | 84 [74-92] | 1,470 (0%) | 83 [74-92] |
|  |  | Cutaneous melanoma | 172 (0%) | 86 [75-93] | 505 (0%) | 86 [76-93] |
|  |  | Head and neck cancer | 227 (0%) | 77 [68-85] | 791 (0%) | 78 [69-86] |
|  |  | Kidney cancer | 88 (0%) | 73 [60-85] | 231 (0%) | 70 [59-81] |
|  |  | Larynx cancer | 162 (0%) | 76 [58-89] | 224 (0%) | 78 [63-90] |
|  |  | Liver cancer | 146 (0%) | 79 [68-89] | 522 (0%) | 79 [68-88] |
|  |  | Lung cancer | 180 (0%) | 80 [72-89] | 716 (0%) | 81 [70-90] |
|  |  | Oesophageal cancer | 867 (0%) | 76 [66-86] | 2,571 (0.1%) | 76 [66-85] |
|  |  | Pancreatic cancer | 1,940 (0%) | 78 [69-88] | 3,639 (0.1%) | 77 [69-87] |
|  |  | Prostate cancer | 216 (0%) | 77 [63-87] | 556 (0%) | 78 [67-87] |
|  |  | Stomach cancer | 45 (0%) | 39 [33-48] | 96 (0%) | 41 [34-51] |
|  |  | Testis cancer | 45 (0%) | 59 [50-76] | 115 (0%) | 60 [48-71] |
|  |  | Thyroid gland cancer | 418 (0%) | 85 [75-93] | 1,190 (0%) | 85 [75-92] |
|  |  | Other or no cancer^a^ | 90,521 (1.9%) | 55 [40-76] | 94,377 (2%) | 55 [40-77] |
|  | Other cause | Bladder cancer | 68,391 (1.4%) | 65 [57-76] | 71,558 (1.5%) | 66 [57-76] |
|  |  | CNS cancer | 23,307 (0.5%) | 68 [58-77] | 24,288 (0.5%) | 69 [59-78] |
|  |  | Colorectal cancer | 145,311 (3%) | 79 [71-88] | 154,505 (3.3%) | 79 [71-88] |
|  |  | Cutaneous melanoma | 45,464 (1%) | 78 [69-88] | 48,924 (1%) | 78 [69-88] |
|  |  | Head and neck cancer | 62,514 (1.3%) | 73 [65-80] | 65,828 (1.4%) | 73 [65-80] |
|  |  | Kidney cancer | 18,149 (0.4%) | 69 [60-79] | 19,322 (0.4%) | 70 [60-79] |
|  |  | Larynx cancer | 13,439 (0.3%) | 80 [71-89] | 13,900 (0.3%) | 81 [72-89] |
|  |  | Liver cancer | 39,944 (0.8%) | 71 [62-81] | 42,045 (0.9%) | 72 [62-81] |
|  |  | Lung cancer | 53,025 (1.1%) | 74 [65-83] | 55,766 (1.2%) | 74 [65-83] |
|  |  | Oesophageal cancer | 263,655 (5.5%) | 70 [62-79] | 275,938 (5.8%) | 71 [62-79] |
|  |  | Pancreatic cancer | 184,993 (3.9%) | 81 [75-90] | 196,328 (4.1%) | 82 [75-91] |
|  |  | Prostate cancer | 31,891 (0.7%) | 77 [67-84] | 33,436 (0.7%) | 77 [68-85] |
|  |  | Stomach cancer | 1,343 (0%) | 43 [33-60] | 1,394 (0%) | 42 [32-58] |
|  |  | Testis cancer | 1,752 (0%) | 74 [64-79] | 1,757 (0%) | 75 [66-80] |
|  |  | Thyroid gland cancer | 77,361 (1.6%) | 81 [73-89] | 81,870 (1.7%) | 81 [73-90] |
|  |  | Other or no cancer^a^ | 3,669,863 (76.5%) | 74 [56-84] | 3,566,184 (74.9%) | 75 [59-85] |
| **Women** | Overall |  | 4,652,949 (100%) | 76 [60-86] | 4,640,867 (100%) | 76 [61-86] |
|  | Suicide | Bladder cancer | 22 (0%) | 67 [56-81] | 70 (0%) | 67 [59-84] |
|  |  | Breast cancer | 15 (0%) | 66 [55-83] | 42 (0%) | 58 [48-78] |
|  |  | CNS cancer | 165 (0%) | 78 [67-88] | 254 (0%) | 77 [67-88] |
|  |  | Colorectal cancer | 20 (0%) | 75 [72-88] | 48 (0%) | 85 [71-94] |
|  |  | Corpus uteri cancer | 15 (0%) | 76 [66-80] | 48 (0%) | 74 [65-80] |
|  |  | Cutaneous melanoma | 4 (0%) | 65 [56-68] | 10 (0%) | 63 [58-66] |
|  |  | Head and neck cancer | 37 (0%) | 66 [52-78] | 58 (0%) | 65 [53-78] |
|  |  | Kidney cancer | 13 (0%) | 77 [63-88] | 33 (0%) | 74 [65-90] |
|  |  | Larynx cancer | 34 (0%) | 73 [60-81] | 91 (0%) | 71 [59-79] |
|  |  | Liver cancer | 42 (0%) | 82 [74-89] | 156 (0%) | 79 [68-88] |
|  |  | Lung cancer | 68 (0%) | 68 [57-78] | 170 (0%) | 70 [57-79] |
|  |  | Oesophageal cancer | 32 (0%) | 79 [67-85] | 75 (0%) | 74 [63-82] |
|  |  | Ovary cancer | 399 (0%) | 63 [52-74] | 617 (0%) | 67 [56-77] |
|  |  | Pancreatic cancer | 45 (0%) | 58 [46-66] | 97 (0%) | 56 [46-67] |
|  |  | Stomach cancer | 55 (0%) | 75 [64-83] | 135 (0%) | 69 [62-80] |
|  |  | Thyroid gland cancer | 20 (0%) | 82 [73-93] | 53 (0%) | 84 [76-94] |
|  |  | Other or no cancer^a^ | 28,866 (0.6%) | 54 [42-70] | 29,112 (0.6%) | 55 [42-70] |
|  | Other cause | Bladder cancer | 21,089 (0.5%) | 69 [58-82] | 21,302 (0.5%) | 69 [58-82] |
|  |  | Breast cancer | 17,310 (0.4%) | 69 [60-79] | 17,527 (0.4%) | 70 [59-79] |
|  |  | CNS cancer | 109,621 (2.4%) | 81 [73-91] | 112,229 (2.4%) | 82 [73-91] |
|  |  | Colorectal cancer | 22,068 (0.5%) | 82 [71-91] | 22,505 (0.5%) | 82 [72-91] |
|  |  | Corpus uteri cancer | 14,771 (0.3%) | 76 [68-85] | 15,052 (0.3%) | 77 [68-85] |
|  |  | Cutaneous melanoma | 2,445 (0.1%) | 68 [59-78] | 2,325 (0.1%) | 68 [59-78] |
|  |  | Head and neck cancer | 11,657 (0.3%) | 79 [67-88] | 12,067 (0.3%) | 79 [67-88] |
|  |  | Kidney cancer | 9,941 (0.2%) | 77 [65-87] | 10,261 (0.2%) | 77 [66-88] |
|  |  | Larynx cancer | 29,969 (0.6%) | 74 [65-82] | 30,660 (0.7%) | 74 [65-83] |
|  |  | Liver cancer | 45,900 (1%) | 78 [69-87] | 46,711 (1%) | 78 [69-88] |
|  |  | Lung cancer | 77,503 (1.7%) | 69 [58-79] | 78,629 (1.7%) | 69 [58-79] |
|  |  | Oesophageal cancer | 14,965 (0.3%) | 78 [70-85] | 15,345 (0.3%) | 78 [69-85] |
|  |  | Ovary cancer | 105,194 (2.3%) | 77 [66-86] | 107,343 (2.3%) | 77 [66-86] |
|  |  | Pancreatic cancer | 3,990 (0.1%) | 73 [63-80] | 4,038 (0.1%) | 73 [62-80] |
|  |  | Stomach cancer | 25,049 (0.5%) | 76 [68-84] | 25,780 (0.6%) | 76 [68-84] |
|  |  | Thyroid gland cancer | 16,388 (0.4%) | 84 [76-92] | 16,783 (0.4%) | 84 [76-92] |
|  |  | Other or no cancer^a^ | 4,095,237 (88%) | 76 [59-85] | 4,071,241 (87.7%) | 76 [60-86] |

CNS: central nervous system; IQR: interquartile range.

^a^ Includes other cancer sites, multiple cancers, and haematological malignancies.

Additional table 3. Suicide ORs by cancer site in men in observed and simulated mortality data, and estimated bias magnitudes

|  | **French mortality data** | |  | **Simulation #1:**  **Independence** | |  | **Simulation #2:**  **RR from Fang *et al.*[4]** | |  | **Fang *et al*. study[4]** | **Collider**  **bias^b^** | **Reporting**  **bias^c^** |
| --- | --- | --- | --- | --- | --- | --- | --- | --- | --- | --- | --- | --- |
| **Cancer site** | **OR** | **[95% CI]** |  | **OR** | **[95% CI]** |  | **OR** | **[95% CI]** |  | **RR** |  |  |
| **Thyroid gland** | 0.06 | [0.02;0.12] |  | 1.26 | [0.94;1.7] |  | 2.98 | [2.46;3.61] |  |  |  |  |
| **No cancer^a^** | 1.00 |  |  | 1.00 |  |  | 1.00 |  |  | 1.0 | 1.0 | 1.0 |
| **Testis** | 0.03 | [0.02;0.06] |  | 0.74 | [0.55;1.00] |  | 1.25 | [1.01;1.54] |  |  |  |  |
| **Prostate** | 0.22 | [0.20;0.23] |  | 0.68 | [0.65;0.71] |  | 1.10 | [1.07;1.14] |  | 1.9 | 1.7 | 5.1 |
| **Cutaneous melanoma** | 0.01 | [0.01;0.02] |  | 0.63 | [0.54;0.74] |  | 0.77 | [0.67;0.88] |  | 1.4 | 1.8 | 59 |
| **Kidney** | 0.06 | [0.05;0.07] |  | 0.36 | [0.32;0.41] |  | 0.82 | [0.75;0.89] |  |  |  |  |
| **Colorectal** | 0.07 | [0.06;0.08] |  | 0.35 | [0.33;0.38] |  | 0.51 | [0.48;0.53] |  | 1.6 | 3.2 | 7.1 |
| **Bladder** | 0.09 | [0.08;0.11] |  | 0.33 | [0.30;0.36] |  | 0.81 | [0.76;0.86] |  |  |  |  |
| **Larynx** | 0.09 | [0.07;0.11] |  | 0.21 | [0.17;0.26] |  | 0.47 | [0.41;0.54] |  |  |  |  |
| **Stomach** | 0.05 | [0.04;0.06] |  | 0.20 | [0.17;0.24] |  | 0.50 | [0.46;0.55] |  |  |  |  |
| **Liver** | 0.02 | [0.02;0.03] |  | 0.19 | [0.17;0.22] |  | 0.58 | [0.54;0.62] |  | 4.5 | 7.8 | 25 |
| **Pancreas** | 0.04 | [0.03;0.05] |  | 0.17 | [0.15;0.20] |  | 0.59 | [0.55;0.64] |  | 4.5 | 7.6 | 15 |
| **Oesophagus** | 0.04 | [0.03;0.05] |  | 0.17 | [0.14;0.20] |  | 0.52 | [0.48;0.57] |  | 4.5 | 8.6 | 13 |
| **Lung** | 0.03 | [0.02;0.03] |  | 0.15 | [0.14;0.16] |  | 0.39 | [0.37;0.41] |  | 3.3 | 8.5 | 14 |
| **Head and neck** | 0.05 | [0.04;0.05] |  | 0.12 | [0.11;0.14] |  | 0.29 | [0.26;0.31] |  |  |  |  |
| **Central nervous system** | 0.01 | [0.01;0.01] |  | 0.11 | [0.09;0.14] |  | 0.24 | [0.21;0.28] |  | 2.3 | 9.5 | 27 |

Logistic regression models adjusted for age (B-spline of degree 3), gender, and region of death; mainland France, 2000-2010.

OR: odds ratio; RR: relative risk; 95% CI: 95% confidence interval.

^a^ Excluding other cancer sites, multiple cancers, and haematological malignancies.

The magnitude of the biases was estimated from the following ratios:

^b^ Collider bias = RR from Fang *et al.* / OR estimated from the data of simulation #2

^c^ Reporting bias = OR estimated from the data of simulation #2 / OR estimated from observed deaths

Additional table 4. Suicide ORs by cancer site in men in observed and simulated mortality data, and estimated bias magnitudes

|  | **French mortality data** | |  | **Simulation #1:**  **Independence** | |  | **Simulation #2:**  **RR from Fang *et al.*[4]** | |  | **Fang *et al.* study[4]** | **Collider**  **bias^b^** | **Reporting**  **bias^c^** |
| --- | --- | --- | --- | --- | --- | --- | --- | --- | --- | --- | --- | --- |
| **Cancer site** | **OR** | **[95% CI]** |  | **OR** | **[95% CI]** |  | **OR** | **[95% CI]** |  | **RR** |  |  |
| **Thyroid gland** | 0.05 | [0.02;0.12] |  | 1.51 | [1.12;2.03] |  | 3.10 | [2.53;3.80] |  |  |  |  |
| **No cancer^a^** | 1.00 |  |  | 1.00 |  |  | 1.00 |  |  | 1.0 | 1.0 | 1.0 |
| **Breast** | 0.03 | [0.03;0.03] |  | 0.56 | [0.50;0.62] |  | 0.81 | [0.75;0.88] |  | 1.6 | 2.0 | 28 |
| **Cutaneous melanoma** | 0.01 | [<.01;0.02] |  | 0.50 | [0.36;0.69] |  | 0.72 | [0.56;0.93] |  | 1.4 | 1.9 | 80 |
| **Corpus uteri** | 0.02 | [0.01;0.03] |  | 0.40 | [0.31;0.53] |  | 0.93 | [0.78;1.10] |  |  |  |  |
| **Kidney** | 0.02 | [0.01;0.04] |  | 0.39 | [0.28;0.56] |  | 0.86 | [0.69;1.09] |  |  |  |  |
| **Colorectal** | 0.02 | [0.01;0.02] |  | 0.31 | [0.27;0.37] |  | 0.46 | [0.40;0.52] |  | 1.6 | 3.5 | 25 |
| **Bladder** | 0.03 | [0.02;0.06] |  | 0.30 | [0.20;0.47] |  | 0.76 | [0.58;0.99] |  |  |  |  |
| **Oesophagus** | 0.02 | [0.01;0.05] |  | 0.20 | [0.12;0.35] |  | 0.48 | [0.34;0.67] |  | 4.5 | 9.4 | 20 |
| **Larynx** | 0.03 | [0.01;0.09] |  | 0.18 | [0.07;0.49] |  | 0.46 | [0.25;0.85] |  |  |  |  |
| **Stomach** | 0.01 | [0.01;0.02] |  | 0.17 | [0.11;0.27] |  | 0.40 | [0.30;0.53] |  |  |  |  |
| **Liver** | 0.01 | [<.01;0.01] |  | 0.17 | [0.10;0.29] |  | 0.53 | [0.40;0.70] |  | 4.5 | 8.5 | 106 |
| **Ovary** | <0.01 | [<.01;0.01] |  | 0.17 | [0.12;0.23] |  | 0.43 | [0.35;0.52] |  |  |  |  |
| **Pancreas** | 0.01 | [0.01;0.02] |  | 0.17 | [0.12;0.22] |  | 0.59 | [0.51;0.69] |  | 4.5 | 7.6 | 49 |
| **Head and neck** | 0.03 | [0.02;0.05] |  | 0.12 | [0.08;0.17] |  | 0.35 | [0.27;0.44] |  |  |  |  |
| **Central nervous system** | <0.01 | [<.01;0.01] |  | 0.10 | [0.06;0.17] |  | 0.27 | [0.20;0.37] |  | 2.3 | 8.5 | 91 |
| **Lung** | 0.01 | [0.01;0.01] |  | 0.10 | [0.08;0.12] |  | 0.23 | [0.19;0.26] |  | 3.3 | 15 | 32 |

Logistic regression models adjusted for age (B-spline of degree 3), gender, and region of death; mainland France, 2000-2010.

OR: odds ratio; RR: relative risk; 95% CI: 95% confidence interval.

^a^ Excluding other cancer sites, multiple cancers, and haematological malignancies.

The magnitude of the biases was estimated from the following ratios:

^b^ Collider bias = RR from Fang *et al.* / OR estimated from the data of simulation #2

^c^ Reporting bias = OR estimated from the data of simulation #2 / OR estimated from observed deaths
